# Elevated neutrophil-to-lymphocyte ratio is an independent poor prognostic factor in patients with intrahepatic cholangiocarcinoma

**DOI:** 10.18632/oncotarget.7680

**Published:** 2016-02-24

**Authors:** Guohe Lin, Yongcheng Liu, Shuhong Li, Yize Mao, Jun Wang, Zeyu Shuang, Jianlin Chen, Shengping Li

**Affiliations:** ^1^ State Key Laboratory of Oncology in South China, Cancer Center, Sun Yat-sen University, Guangzhou, China; ^2^ National Collaborative Innovation Center for Cancer Medicine, Guangzhou, China; ^3^ Department of Hepatobiliary Oncology, Sun-Yat-sen University Cancer Center, Guangzhou, China; ^4^ Department of Surgical Oncology, Sir Run Run Shaw Hospital, Zhejiang University School of Medicine, Hangzhou, China; ^5^ Department of Endoscopy, Sun Yat-sen University Cancer Center, Guangzhou, China

**Keywords:** intrahepatic cholangiocarcinoma, neutrophil-to-lymphocyte ratio, CD4+ T cells, CD8+ T cells, prognosis

## Abstract

We investigated whether elevated neutrophil-to-lymphocyte ratio (NLR) was associated with poor anti-tumor immunity and prognosis in patients with intrahepatic cholangiocarcinoma (ICC). Clinicopathologic data of 102 patients with ICC who underwent hepatectomy was retrospectively analyzed. The Kaplan-Meier method and Cox regression model were used to analyze the survival and prognosis. The percentage of overall lymphocytes, T cells and CD8+ T cells in the high NLR group was lower than that in the low NLR group. The percentage of PD-1+CD4+ and PD-1+CD8+ T cells was higher and the percentage of IFN-γ+CD4+ and IFN-γ+CD8+ T cells was lower in the high NLR group than that in the low NLR group (*p* = 0.045, *p* = 0.008; *p* = 0.012, *p* = 0.006). Density of tumor-infiltrating CD3+ T cells in the high NLR group was lower than that in the low NLR group (*p* < 0.001). Elevated NLR was an independent predictor for poor overall survival (OS; *p* = 0.035) and recurrence-free survival (RFS; *p* = 0.008). These results indicate that elevated NLR is associated with poor anti-tumor immunity and could be a poor biomarker for prognosis in patients with ICC.

## INTRODUCTION

Intrahepatic cholangiocarcinoma (ICC) and hepatocellular carcinoma (HCC) are the most common primary liver tumors, but ICC is a fatal malignancy which arises from the epithelium of intrahepatic bile duct [1]. Currently, surgical resection remains the only effective treatment for early stage ICC, but most patients lose the chances because effective methods for diagnosis of the disease in early stage are lacking [2]. Adjuvant chemotherapy and radiotherapy have shown limited effects for patients with ICC because of insensitivity to chemotherapeutics and resistance to radiotherapy [3–4]. The 5 years survival rate of patients with ICC is only 2.6% [5].

The inflammatory response seems to contribute to the occurrence and development of numerous cancers [6]. Patients with primary sclerosing cholangitis characterized by chronic biliary inflammation have a high risk of developing to biliary tract cancers [7]. However, immune cells such as lymphocytes can contribute to or inhibit the development of tumors depending on the cytokines or effective molecules in the tumor microenvironment [8, 9]. CD8+ T cells play a vital role in preventing the development of tumors. However, PD-1 expression on CD8+ T cells is upregulated in patients with HCC and activation of PD-1 is an important mechanism by which tumor cells can inhibit anti-tumor immune responses mediated by CD8+ T cells [10, 11]. Thus, both the inflammatory response and immune response participate in the process of tumor development.

Several studies have shown that a high NLR is a prognostic factor for poor OS and RFS in many types of cancers, including colorectal cancer and diffuse large B-cell lymphoma [12, 13]. Recently, studies have shown that NLR reflects the balance between the inflammatory response and the immune response, but these studies did not analyze the relationship between NLR and anti-tumor immunity [14, 15]. Our study was designed to evaluate whether elevated NLR was associated with poor anti-tumor immunity and was a poor biomarker for OS and RFS in patients with ICC.

## RESULTS

### The clinic and pathologic features of ICC patients with high and low NLR

The patients with ICC were divided into high NLR group (*n* = 43) and the low NLR group (*n* = 59) according to the cutoff of 3. There were no statistic differences between high and low NLR patients in age, gender, lymph node metastases, liver cirrhosis, distant metastases, tumor differentiation or tumor number (Table 1). Elevated NLR was associated with elevated plasmic CEA (*p* = 0.033; Table 1). Tumor size in the high NLR group was significantly larger than that in the low NLR group (*p* = 0.024; Table 1). Moreover, NLR increased progressively through early TNM stage to advanced TNM stage (*p* = 0.015; Table 1). Our results indicated that elevated NLR was associated with the development and progression of ICC.

**Table 1:** The correlation between NLR and clinicopathological characteristics in patients with ICC

Factors	NLR < 3	NLR ≥ 3	*P*
**Gender**			
Male	34	32	
Female	25	11	0.0611
**Age (years)**			
≤ 50	20	15	
>50	39	28	0.541
**CA19-9 (U/ml)**			
< 35	27	15	
≥ 35	32	28	0.185
**CEA (ng/ml)**			
< 5	49	28	
≥ 5	10	15	**0.033**
**LDH (U/ml)**			
< 206	33	30	
≥ 206	26	13	0.112
**GGT (U/ml)**			
< 46.3	8	8	
≥ 46.3	51	35	0.336
**AFP (ng/ml)**			
< 25	54	40	
≥ 25	5	3	0.544
**Lymph node metastases**			
Yes	16	16	
No	43	27	0.192
**Tumor size (cm)**			
≤ 5	32	14	
> 5	27	29	**0.024**
Tumor number			
Single	44	28	
Multiple	15	15	0.207
**Distant metastases**			
Yes	3	6	
No	56	37	0.115
**Tumor differentiation**			
I–II	27	19	
III–IV	32	24	0.518
**Liver cirrhosis**			
Yes	17	12	
No	42	31	0.550
**TNM staging**			
I–II	36	16	
III–IV	23	27	**0.015**

### Elevated NLR was an independent prognostic factor for poor overall survival

The OS of the patients in the high NLR group was significantly shorter than that in the low NLR group (*p* = 0.009; Figure 2A). Univariate analysis identified lymph node metastases (*p* < 0.001), tumor number ≥ 2 (*p* = 0.002), tumor size ≥ 5cm (*p* = 0.006), advanced TNM stage (*p* < 0.001) and NLR ≥ 3 (*p* = 0.010) as significant prognostic factors for poor OS (Table 2). Multivariate analysis identified lymph node metastases (*p* = 0.011) and NLR ≥ 3 (*p* = 0.035) as independent prognostic factors for poor OS (Table 2).

**Table 2:** Univariate and multivariate analysis of the associations between prognostic variables and overall survival in patients with ICC

Variables	Univariate			Multivariate		
	HR	95% CI	*P*	HR	95% CI	*P*
**Age** (years, > 50 *vs*. ≤50)	1.217	0.707-2.097	0.478			
**Gender** (female *vs*. male)	0.760	0.441-1.308	0.321			
**CA 19-9** (U/ml, ≥ 35 *vs*. < 35)	1.147	0.687-1.914	0.601			
**CEA** (ng/ml, ≥ 5 *vs*. < 5)	1.076	0.600-1.933	0.600			
**LDH** (U/ml, ≥ 206 *vs*. < 206)	1.217	0.734-2.018	0.447			
**GGT** (U/ml, ≥ 46.3 *vs*. < 46.3)	1.711	0.775-3.777	0.183			
**AFP** (ng/ml, ≥ 25 *vs*. < 25)	1.528	0.657-3.556	0.325			
**Lymph node metastases** (yes *vs*. no)	2.854	1.711-4.759	**<0.001**	2.103	1.182-3.741	**0.011**
**Tumor size** (cm, ≥ 5 *vs*.< 5)	2.086	1.239-3.512	**0.006**	1.385	0.793-2.420	0.252
**Tumor number** (multiple *vs*. single)	2.274	1.356-3.813	**0.002**	1.641	0.937-2.875	0.083
**Distant metastases** (yes *vs*. no)	1.853	0.880-3.902	0.104			
**Tumor differentiation** (I-II *vs*.III-IV)	1.272	0.763-2.121	0.357			
**Liver cirrhosis**(yes *vs*. no)	1.621	0.953-2.756	0.075			
**TNM stage** (I-II *vs*.III-IV)	3.104	1.821-5.293	**<0.001**	1.765	0.923-3.374	0.086
**NLR** (≥ 3 *vs*. < 3)	1.951	1.174-3.242	**0.010**	1.756	1.040-2.963	**0.035**

**Abbreviations:** CEA, carcino-embryonicantigen; CA19-9, carbohydrate antigen 19-9, HR, hazard ratio; CI, confidence interval

### Elevated NLR was an independent prognostic factor for poor recurrence-free survival

The RFS in the high NLR group was significantly shorter than that in the low NLR group (*p* = 0.015; Figure 2B). Univariate analysis identified tumor number ≥ 2 (*p* = 0.004), liver cirrhosis (*p* = 0.030), AFP ≥ 25 ng/ml (*p* = 0.002) and NLR ≥ 3 (*p* = 0.017) as significant prognostic factors for poor RFS (Table 3). Multivariate analysis identified tumor number ≥ 2 (*p* = 0.001), liver cirrhosis (*p* = 0.045), AFP ≥ 25 ng/ml (*P* = 0.005) and NLR ≥ 3 (*p* = 0.008) as independent prognostic factors for poor RFS (Table 3).

**Table 3:** Univariate and multivariate analysis of the associations between prognostic variables and recurrence-free survival in patients with ICC

Variables	Univariate			Multivariate		
	HR	95% CI	*P*	HR	95% CI	*P*
**Age** (years, > 50 *vs*. ≤50)	1.039	0.569-1.902	0.901			
**Gender** (female *vs*. male)	0.630	0.340-1.165	0.141			
**CA 19-9** (U/ml, ≥ 35 *vs*. < 35)	0.835	0.471-1.482	0.539			
**CEA** (ng/ml, ≥ 5 *vs*. < 5)	1.745	0.932-3.267	0.082			
**LDH** (U/ml, ≥ 206 *vs*. < 206)	1.089	0.607-1.953	0.774			
**GGT** (U/ml, ≥ 46.3 *vs*. < 46.3)	1.201	0.537-2.687	0.656			
**AFP** (ng/ml, ≥ 25 *vs*. < 25)	3.655	1.628-8.208	**0.002**	3.713	1.502-9.181	**0.005**
**Lymph node metastases** (yes *vs*. no)	1.285	0.672-2.457	0.449			
**Tumor size** (cm, ≥ 5 *vs*.< 5)	1.725	0.962-3.091	0.067			
**Tumor number** (multiple *vs*. single)	2.406	1.329-4.356	**0.004**	2.782	1.494-5.182	**0.001**
**Distant metastases** (yes *vs*. no)	1.697	0.670-4.299	0.265			
**Tumor differentiation** (I-II *vs*.III-IV)	1.617	0.892-2.932	0.114			
**Liver cirrhosis**(yes *vs*. no)	1.966	1.068-3.618	**0.030**	2.016	1.017-3.997	**0.045**
**TNM stage** (I-II *vs*.III-IV)	1.515	0.848-2.707	0.160			
**NLR** (≥ 3 *vs*. < 3)	2.009	1.131-3.571	**0.017**	2.260	1.242-4.114	**0.008**

**Abbreviations:** CEA, carcino-embryonicantigen; CA19-9, carbohydrate antigen 19-9, HR, hazard ratio; CI, confidence interval.

### Elevated NLR was associated with poor anti-tumor immunity

**Figure 1:**
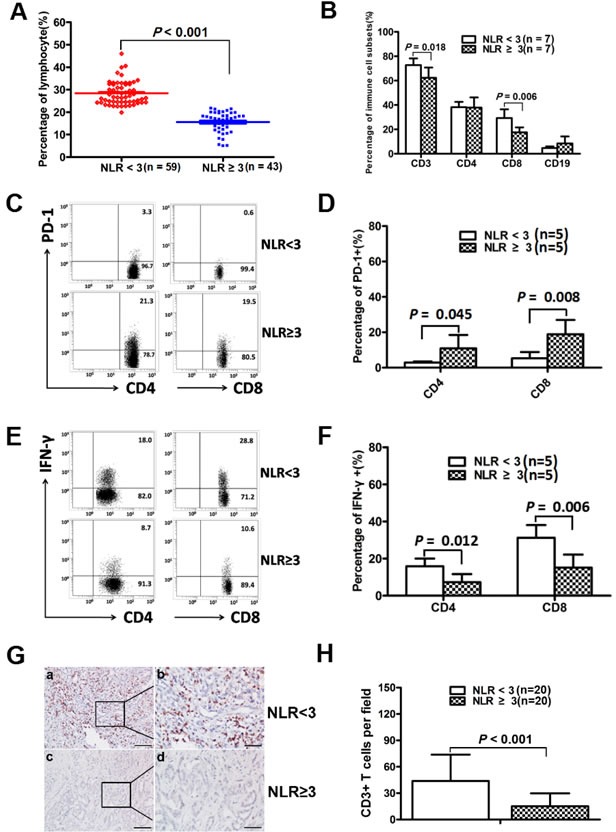
Elevated NLR was associated with poor anti-tumor immunity **A.** The percentage of overall lymphocytes to leukocytes in the high NLR group was significantly lower than that in the low NLR group (*p* < 0.001). **B.** The percentage of T cells and CD8+ T cells to total lymphocytes in the high NLR group was significantly lower than that in the low NLR group (*p* = 0.018; *p* = 0.006). **C.**, **D.** The percentage of PD-1+CD4+ and PD-1+CD8+ T cells in the high NLR group were higher than that in the low NLR group (*p* = 0.045, *p* = 0.008, *n* = 5). **E.**, **F.** The percentage of IFN-γ+CD4+ and IFN-γ+CD8+ T cells in the high NLR group were lower than in the low NLR group (*p* = 0.012, *p* = 0.006, *n* = 5). **G.** Representative immunohistochemistry images of CD3 in intratumoral regions. Scale bar, 100 μm (a, c), 50μm(b, d). **H.** The numbers of CD3+ T cells in the high NLR group and in the low NLR group. Cell numbers were calculated as the cell count per ×400 field.

The percentage of overall lymphocytes to leukocytes in the high NLR group was lower than that in the low NLR group (15.59 ± 4.60 *vs*. 28.44 ± 5.20, *p* < 0.001; Figure 1A). Furthermore, we found that the percentage of T cells and CD8+ T cells to total lymphocytes in the high NLR group was lower than that in the low NLR group (62.33 ± 8.36 *vs*. 72.73 ± 5.50, *p* = 0.018; 17.51 ± 4.11 *vs*. 29.26 ± 7.18, *p* = 0.006, *n* = 7; Figure 1B). There were no significant differences in the percentage of CD4+ T cells and B cells to total lymphocytes between the high NLR group and the low NLR group (38.20 ± 4.38 *vs*. 37.90 ± 8.32, *p* = 0.934; 4.71 ± 1.31 *vs*. 8.49 ± 5.71, *p* = 0.113, *n* = 7; Figure 1B). The percentage of PD-1+CD4+ and PD-1+CD8+ T cells in the high NLR group was higher than that in the low NLR group (10.92 ± 7.56 *vs*. 2.88 ± 0.63, *p* = 0.045; 18.90 ± 8.04 *vs*. 5.28 ± 3.54, *p* = 0.008, *n* = 5; Figure 1C, 1D). Compared with the low NLR group, the percentage of IFN-γ+CD4+ and IFN-γ+CD8+ T cells was lower in the high NLR group (7.30 ± 4.31 *vs*. 15.92 ± 4.13, *p* = 0.012; 15.16 ± 7.01 *vs*. 31.22 ± 6.87, *p* = 0.006, *n* = 5; Figure 1E, 1F). The density of tumor-infiltrating CD3+ T cells in the high NLR group was lower than that in the low NLR group (15 ± 14 *vs*. 43 ± 30 cells/field, *p* < 0.001, *n* = 20; Figure 1G, 1H). Our results indicated that elevated NLR was associated with poor anti-tumor immunity.

**Figure 2:**
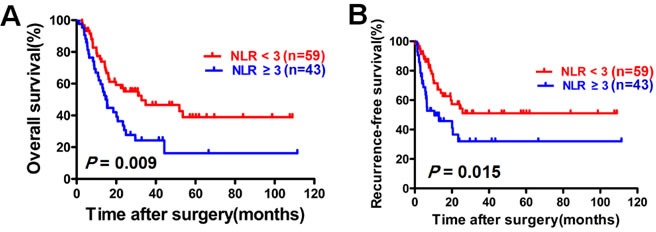
Elevated NLR correlates with a poor prognosis in patients with ICC The patients with ICC were divided into high NLR group (*n* = 43) and the low NLR group (*n* = 59) according to the cutoff of 3. Kaplan-Meier analysis was conducted to disclose the relationship of NLR with the overall survival **A.** and recurrence-free survival **B.** (log-rank test).

## DISCUSSION

Excessive inflammation contributes to the development of tumor cells [16]. Neutrophils contribute to the process of inflammation by activating proangiogenic factors including vascular endothelial growth factor or inflammatory cytokines including IL-1β [17–18]. Elevated neutrophil levels are associated with decreased OS in advanced non-small-cell lung cancer [19]. Poor anti-tumor immunity in the tumor microenvironment is another important factor for tumor progression. Lymphocytes are crucial components of innate immunity and the adaptive immune response, and can eradicate tumor cells by cytotoxic cell death and cytokine secretion [10]. Some evidence shows that a low lymphocyte count could be responsible for a weakened defense against cancers [20]. A low density of CD8+ T cells in tumor tissues is associated with poor prognosis in patients with hepatocellular cancer [21, 22]. In addition, peripheral blood neutrophils suppress the cytolytic activity of lymphocytes and natural killer cells to tumor cells *in vitro* [23].

The dysfunction of CD8+ T cells is an important factor for poor anti-tumor immunity in tumor progression. Some underlying mechanisms for this dysfunction are attributed to increased levels of immunosuppressive cytokines or molecules such as TGF-β, PD-1 and Tim3 [24–27]. Recent researches indicate that NLR reflects the balance between the inflammatory response and the immune response, but these studies do not analyze impaired immune cell subsets or the potential mechanisms [14, 15]. We found that elevated NLR was associated with poor anti-tumor immunity in patients with ICC. First, the percentage of overall lymphocytes to leukocytes in the high NLR group was lower than that in the low NLR group. Second, the percentage of T cells and CD8+ T cells to total lymphocytes in high NLR group was lower than that in the low NLR group. Third, we found that the percentage of PD-1+CD4+ and PD-1+CD8+ T cells was higher and the percentage of IFN-γ+CD4+ and IFN-γ+CD8+ T cells was lower in the high NLR group than that in the low NLR group. The results indicated that elevated NLR was associated with increased PD-1 expression and decreased IFN-γ secretion. Last, elevated NLR was associated with reduced tumor-infiltrating CD3+ T cells in tumor tissue. Our results indicate that the poor prognosis in high NLR group could be ascribed to an excessive inflammatory response and poor anti-tumor immunity response.

Recently, increasing evidence has shown that elevated NLR is associated with poor clinical outcome in patients with various cancers [28–30]. Chen *et al.* showed that elevated NLR predicted a poor prognosis in patients with intrahepatic cholangiocarcinoma who underwent hepatectomy [31]. These findings are in line with our results. In our study, the optimal cutoff of 3 was used for NLR because of its convenience and powerful prognostic value [32, 33]. We found that elevated NLR was associated with decreased OS and RFS. Our results show that NLR ≥ 3 is an independent predictor for poor OS and RFS, which indicates that elevated NLR could be a convenient biomarker to identify patients with a poor prognosis after liver resection.

There were some limitations in this study. First, this was a retrospective analysis from a single center. Second, we did not analyze preoperative C reactive protein (CRP) change together with NLR because it is not routinely measured in our daily practice. Third, immunosuppressive cells such as regulatory T cell (Treg) were not included in this study. Treg can contribute to the development of cancer by inhibiting the proliferation of T cells and secreting suppressive cytokines [34–36].

In conclusion, our study demonstrates that elevated NLR is associated with poor anti-tumor immunity and could be used to identify patients with a poor prognosis. Elevated NLR is a poor biomarker for OS and RFS in patients with ICC after hepatectomy.

## MATERIALS AND METHODS

### Patients and tissue specimens

102 consecutive patients were diagnosed with ICC and received liver resection in the Department of Hepatobiliary Surgery at the Cancer Center of Sun Yat-sen University (Guangzhou, China) between November 1999 and November 2011. None of patients underwent preoperative therapies such as radiotherapy or chemotherapy. Neutrophil and lymphocyte counts were obtained 1 week before histologically proven diagnosis. NLR was calculated by dividing the absolute neutrophil count by the absolute lymphocyte count. Applying receiver operating characteristic(ROC) curve, the cutoff for the NLR was 2.96, but 3 was used for the optimal cutoff for the NLR because of its convenience and powerful prognostic value (NLR < 3, NLR ≥ 3) [32, 33]. Follow-up of patients was performed every three months in the first 3 years and 6 months thereafter. OS was defined as the interval between surgery and death or between surgery and the last observation for surviving patients. RFS was defined as the interval between surgery and recurrence or between surgery and the last observation for patients without recurrence. The research was approved by the institutional review board of Sun Yat-sen University Cancer Center, and written informed consent was obtained from each patient involved in the study.

### Immunohistochemistry

5-μm thick formalin-fixed and paraffin-embedded samples were processed for immunohistochemistry stainning with antibodies against human CD3 (1:500 dilution, Dako A/S, Glostrup, Copenhagen, Denmark) or control antibodies (Santa Cruz Biotechnology, Santa Cruz, CA, USA). The density of CD3+ were evaluated quantitatively by the mean number at five representative fields at 400× magnification by two independent observers who were blinded to the clinical outcome. A significant linear correlation existed between the counts of two independent observers and the average counts of the two investigators was used in subsequent analyses to minimize inter-observer variability.

### Flow cytometry analysis

Peripheral blood mononuclear cells (PBMCs) were isolated from fresh heparinized blood by Ficoll density gradient centrifugation. For phenotypic analysis, the cells were washed twice and stained for 30 min on ice with mixtures of fluorescence-conjugated surface mAbs or isotype-matched controls, and the cells were then washed twice and resuspended in PBS buffer for flow cytometry analysis. PBMCs were stained with the following antibodies: phycoertthrin-cyanin 5 (PE-Cy5)-conjugated anti-CD3 (eBioscience, San Diego, CA, USA); fluorescein isothiocyanate (FITC)-conjugated anti-CD4, anti-CD8 and anti-CD19 (eBioscience); phycoertthrin-cyanin7 (PE-Cy7)-conjugated anti-CD4 (eBioscience); PE-conjugated anti-PD-1 (eBioscience) and anti-IFN-γ (eBioscience).

### Statistical analysis

The optimal cutoff value for NLR was determined using time-dependent ROC curve analysis, which was performed using R software, version 3.2.2. (The R foundation for statistical computing, Vienna, Austria. http://www.r-pro-ject.org) and the ‘survival ROC’ package. Statistical analysis was performed using SPSS_13.0 statistical software. The differences in numerical data between the two groups were compared by Student *t* tests. Categorical variables were compared using the *χ^2^*-tests. The Kaplan-Meier method and Cox regression model were used to analyze survival and prognosis. All statistical tests were two-sided, and a significant difference was considered when *p* < 0.05.
